# Book Reviews

**Published:** 1875-12

**Authors:** 


### BOOK REVIEWS.

NEARER MY GOD TO THEE.

Every body who appreciates this beautiful hymn—and who, that has seen it, does not?—will be delighted to preserve it in so attractive a form, as published this year, by Lee and Shepherd, of Boston. The sweet face of the writer, Miss Sarah Flower Adams, appears in vignette, and a charming Easter cross embellishes the title page, while the rest of the illustrations exhibit great feeling and artistic skill. These are designed by Miss S. B. Humphrey, and the lovely poems, so appropriately adorned, will make one of the most welcome Christmas presents of the season.

THE RIVER OF DREAMS. By G. E. O. Author of "Thurid, and other Poems."

THIS is a nicely gotten up little book of poems, from the first of which the title is taken. This is a delicate, dreamy production, well calculated to please lovers of the imaginative. From the miscellaneous poems which complete the volume, we select the following Sonnet:

That man alone is king who nothing fears;
Who rests, and waits with ever tranquil [heart
And soul, where never, paling doubt uprears
Its aimless face, to bear his destined part;
Who live the present bravely, nor doth start
With blanching cheek, when through the mist [of years,
Vague phamtons of a future dimmed with tears,
Stalk sorrowing to greet him as he nears;
Dull harmless shades which ever trembling bow
Before that master-will and dauntless brow
Which speak the majesty of self-wrought power.
With this broad empire doth each one endow
Himself, alone, who sees misfortune lower,
Unmoved, and holds his sceptre from that hour.

GOING WEST. By Oliver Optic.

The boys will be gladdened yet again by a book from their favorite author, who is so well known that it seems almost superfluous to say one word in commendation. It will be sufficient therefore to state that this volume, though the story is complete in itself, is the first of a series dedicated to the Boys of the Great West, and is the fifty-third book, written by Oliver Optic, especially to please and instruct our young people.

All of the above are published by Lee and Shepherd, Boston Mass.

